# Immunotherapy and NSCLC: The Long and Winding Road

**DOI:** 10.3390/cancers12092512

**Published:** 2020-09-04

**Authors:** Antonio Rossi

**Affiliations:** Therapeutic Science and Strategy Unit, IQVIA, Via Fabio Filzi 29, 20124 Milan, Italy; arossi_it@yahoo.it or antonio.rossi@iqvia.com; Tel.: +39-02-697861

Lung cancer is one of the most incident tumors worldwide, characterized by a very bad prognosis due to its high mortality even in early stages. It was predicted that the new lung cancer cases were about 2.1 million with around 1.8 million deaths every year, worldwide [1,2]. These data have led to lung cancer being considered as a “big killer” [3].

The field in which the match against non-small-cell lung cancer (NSCLC) is played with greater intensity is the metastatic disease. It is precisely in this setting where many steps forward have been made in preclinical and clinical research. In the past century, lung tumor morphology, i.e., small-cell lung cancer (SCLC) or NSCLC, drew the choice of systemic therapy. At the beginning of this century the diagnosis of NSCLC histotype was mandatory to address the correct chemotherapy regimen and later on, the tumor genomics became of paramount importance for the appropriate treatment choice. In fact, deepening of the knowledge of intrinsic mechanisms of cancer growth has led to the discovery of specific pathways and potential targets to focus on. The following development of corresponding drug inhibitors changed the natural history of these NSCLC subtypes in selected subgroups of patients [4]. Then, the cancer cell was no longer the only target changing the paradigm: the immune system became another field of research. The programmed death ligand-1 (PD-L1) tumor proportion score (TPS) expression ≥50%, detected in about 25–30% of NSCLC patients, identifies a subgroup that can benefit from first-line single-agent pembrolizumab, an immunoglobulin (Ig) G4 anti-PD-1 (programmed cell death-1) monoclonal antibody (mAb). Moreover, the combinations of immunotherapy plus platinum-based regimens are also available in the clinical practice for the first-line treatment of NSCLC patients, regardless of PD-L1 expression [5,6].

Overall, to date, before defining the best strategic approach for a patient with metastatic NSCLC, a deeper knowledge of the required baseline characteristics of the disease of that specific patient is mandatory.

So far, one mechanism of immune escape changing paradigm in the management of metastatic NSCLC is through the modulation of immune checkpoints, including cytotoxic T-lymphocyte antigen 4 (CTLA-4) and the axis PD-1/PD-L1, which regulate the priming phase and effector phase of T-cell activation, respectively. mAbs inhibiting these immune checkpoints promote antitumor immunity and can produce durable cancer regression [7].

In order to optimize the outcomes of immunotherapy and costs, biomarkers that predict response have been evaluated to guide treatment decisions in the clinical practice. PD-L1 expression by immunohistochemistry (IHC) is currently used, due to its predictive utility in prospective clinical trials, as a validated biomarker to select patients with metastatic NSCLC who could benefit from pembrolizumab treatment. IHC is approved by the regulatory agencies as a companion diagnostic test prior to immunotherapy [8]. More recently, clinical trial data showed the utility of measuring microsatellite instability (MSI) status and/or DNA mismatch repair deficiency (dMMR) as predictive markers for response to PD-1 blockade by pembrolizumab, independently of tumor cell of origin, resulting in the first Food and Drug Administration (FDA) pan-cancer approval of a therapeutic in oncology [9]. The tumor mutational burden (TMB) includes the total number of mutations detected in tumor cells evaluated by the number of mutations (synonymous and non-synonymous) per megabase based on new generation sequencing (NGS) technologies using whole exome sequencing (WES) or large NGS panels [10]. However, TMB use has several limitations related to its characteristics as a dynamic biomarker with spatial and temporal heterogeneity, the reproducibility of the technique, and the costs.

Due to the complexity of tumor-immune interactions, the described biomarkers appear to be surrogate in the prediction of response to checkpoint inhibitors yielding limited and incomplete information on the complex and dynamic nature of the tumor-immune relationships. Thus, studies to detect further potential predictive biomarkers correlated to immunotherapy outcomes are necessary.

Despite the fact that durable response rates are observed in about one/third of metastatic NSCLC patients treated with immunotherapy, other patients do not benefit from the treatment. Several mechanisms are responsible for the resistance to immunotherapy, which identify three different types. The primary resistance is defined as an absence of response to immunotherapy; the adaptive resistance, in which cancer cells are recognized by the immune system but escape from immune attack; and, after a variable period of activity of immunotherapy, an acquired resistance may onset. The primary and/or adaptive resistances recognize tumor cell-intrinsic factors, including the expression or repression of genes and pathways in tumor cells that prevent immune cell infiltration or function within the tumor microenvironment. In these mechanisms there have been identified: the enhancement of phosphoinositide 3-kinases (PI3K) signaling pathway; the expression of WNT/β-catenin signaling pathway; loss of interferon-gamma (IFNγ) signaling pathways; and lack of T-cell responses due to the loss of tumor antigen expression. Tumor cell-extrinsic mechanisms that lead to primary and/or adaptive resistance involve components within the tumor microenvironment, including regulatory T-cells (Tregs), myeloid derived suppressor cells (MDSCs), M2 macrophages and other inhibitory immune checkpoints. The potential mechanisms of acquired resistance include the loss of T-cell function, lack of T-cell recognition by downregulation of tumor antigen presentation, and development of escape mutation variants in the cancer [11].

The immune world is a very complex system in which several factors are involved that can influence the effect of immunotherapy: microenvironment and environmental factors, internal microflora, heterogeneity of intratumor neoantigens. These factors have to be considered also in the view of overcoming the resistance to immunotherapy [12]. Several efforts are being performed to overcome this resistance, starting from the evidence of existing “hot” tumors, so called because they are T-cell infiltrated and inflamed and most likely to respond to immunotherapy. On the contrary, “cold” tumors are characterized by low immune infiltrates and have not been recognized by the immune system, therefore have not been penetrated by T-cells with the tumor microenvironment enriched by myeloid-derived suppressor cells (MDSC) and regulatory suppressive Tregs, leading to an “immune desert” around the tumor. The knowledge of mechanisms responsible for “hot” and “cold” immune tumors is critical to overcome primary, adaptive, and acquired resistance. Several potential approaches, such as to prime or enhance T-cell responses (e.g., vaccines or adoptive T-cell transfer) with the concurrent removal of co-inhibitory signals (e.g., immune checkpoint inhibitors or MDSC depletion) and/or providing co-stimulatory signals (e.g., anti-OX40), are being investigated to transform immunologically “cold” into “hot” tumors to increase the chances of immunotherapy effectiveness [13,14]. Moreover, the use of chemotherapy and radiotherapy, by inducing immunogenic cell death, or by stimulating innate immune responses and dendritic cell function (e.g., type I-IFN, Toll-like receptor ligands, and oncolytic viruses) can also contribute in promoting the formation or presentation of suitable neoantigens in tumors with a non-inflamed, non-immune-cell infiltrated tumor microenvironment [15]. Additionally, epigenetic modifications of cancer DNA, causing changes in immune-related gene expression, may play a role by enabling the re-expression of immune-related genes, with the potential for therapeutic impact, especially in combination with immunotherapy [16].

The tumor microenvironment, including tumor cells, peripheral immune cells, neovascularization, endothelial cells, fibroblasts, and extracellular matrix, also contribute to the efficacy of immunotherapy [17]; moreover, the number and type of intestinal flora affect not only the incidence of cancer but also the sensitivity to chemotherapy and immunotherapy [18].

Looking to the future, the management of metastatic cancers, and particularly NSCLC, will evaluate the combination of several drugs, such as chemotherapeutics, targeted agents (i.e., biomarkers and anti-angiogenic small molecules and mAbs), immunotherapeutics (i.e., mAbs directed against all the potential factors involved in the immune system), in order to inhibit all the possible targets, also enhancing all the potential physiologic defenses. These forthcoming combination regimens, contributing to the continuous changing of the paradigm shift in the treatment of advanced NSCLC as has happened over the last decades (Figure 1), could influence the design of the next clinical trials; however, the safety and quality of life of patients should be taken into account.

In this view, drugs able to inhibit more targets simultaneously present a further step towards the optimization of the therapeutic strategy in the fight against NSCLC. In addition, among these new generation drugs, M7824 (MSB0011359C), bintrafusp alfa, is an innovative first-in-class bifunctional fusion protein consisting of a human IgG1 mAb against PD-L1 fused to the extracellular domain of transforming growth factor-beta receptor II (TGF-βRII) to function as a TGFβ “trap” [19]. In fact, interesting results were reported by a phase one, open-label trial, including 80 advanced previously-treated NSCLC patients randomized to receive bintrafusp alfa at either 500 mg or the recommended phase two dose of 1200 mg every two weeks. The objective response rate (ORR), primary endpoint, in all patients was 21.3%, while it was 17.5% and 27.5% for the 500 mg dose and the 1200 mg dose, respectively. At the 1200 mg dose, patients with PD-L1-high expression (≥80% expression on tumor cells with Ab clone 73-10, which is equivalent to ≥50% with 22C3) had an ORR of 85.7%. In the 40 patients receiving bintrafusp alfa 1200 mg, the 18 and 24 month progression-free survival (PFS) and overall survival (OS) rates were 18.4% and 11.0%, and 49.7% and 39.7%, respectively. Median OS in patients with positive PD-L1 expression (≥1%) was 21.7 months. Grade ≥3 treatment-related adverse events occurred in 29% of patients and led to treatment discontinuation in 10% of cases [19,20]. To date, bintrafusp alfa is being investigated, within randomized trials (NCT03631706, NCT03840902), for the management of advanced NSCLC patients.

Only a deeper knowledge of the mechanisms underlying the relationships between cancer and host, including the interplay of the human immune system, which is a complicated mix of many different cells, tissues and chemical factors that work together to try to maintain health, can improve our ability to reach the goal of turning NSCLC into a chronic disease. All efforts made so far are to be considered as a first step towards the “precision medicine” defined as the right drug to the right patient but also at the right moment. In fact, considering the unpredictability of NSCLC, at metastatic stage first diagnosis, a complete profiling of the disease is needed to start the most appropriate and effective approach as soon as possible.

Overall, despite the road still being long and winding, immunotherapy is a great breakthrough to current, existing tumor treatment, and a promising new type of advanced cancer treatment, particularly for NSCLC, which is facing great challenges and opportunities in a path that will surely develop positively.

## Figures and Tables

**Figure 1:**
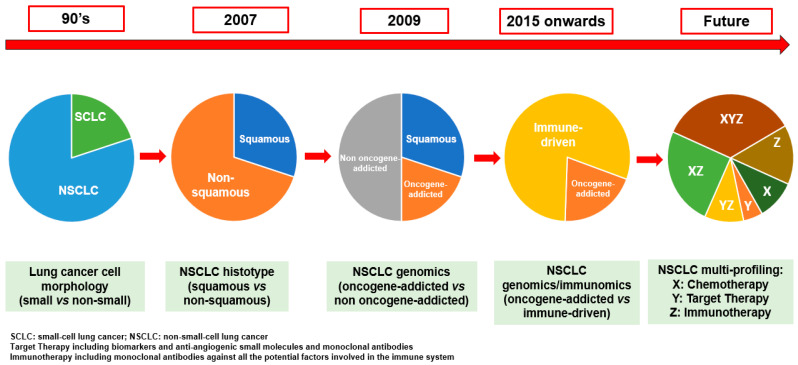
The evolution of paradigm shift of first-line treatment of fit metastatic non-small-cell lung cancer (NSCLC) patients.
